# The Embolization of the Segmental Branch of Hepatic Artery Pseudoaneurysm Following Endoscopic Retrograde Cholangiopancreatography: A Case Report

**DOI:** 10.7759/cureus.64178

**Published:** 2024-07-09

**Authors:** Hafiz Haseeb, Irfan Ullah, Jamal Ahmed, Mansoor Zafar

**Affiliations:** 1 Gastroenterology, The Grange University Hospital, Cwmbran, GBR; 2 Gastroenterology and Hepatology, The Grange University Hospital, Cwmbran, GBR; 3 Gastroenterology, University Hospital Llandough, Penarth, GBR; 4 Gastroenterology, Hammersmith Hospital, Imperial College Healthcare NHS Trust, London, GBR

**Keywords:** upper gastrointestinal(ugi) bleeding, pseudoaneurysm, interventional radiology guided embolization, computed tomography abdomen, endoscopy ercp

## Abstract

Endoscopic retrograde cholangiopancreatography (ERCP) remains the main therapeutic modality towards the management of common bile duct (CBD) stones and dilatation of strictures. It also has varied diagnostic roles including brush biopsy. The procedure still is associated with side effects and increased morbidity and mortality. One side effect is bleeding. This may be associated with procedural trauma or bleeding following post-traumatic pseudoaneurysm delayed-onset bleeding. Although it may be argued that inflammation surrounding the biliary duct area and in particular the pancreas could also contribute to the delayed bleeding along the ampullary region, we present a case of delayed pseudoaneurysm bleeding that was successfully managed post-ERCP via interventional radiology-guided embolization.

## Introduction

Despite the advancement in technologies and profound operator skills acquired through intense training experience, endoscopic retrograde cholangiopancreatography (ERCP) remains to be associated with a high risk for complications. The most reported risk factors are perforation, bleeding, cholangitis, and pancreatitis [1].

Pancreatitis remains one of the most associated risk factors with ERCP, and the reported incidence of pancreatitis following ERCP ranges from 1% to 6% in the general population and up to 30% in the high-risk population [2]. Mechanical trauma and contrast injection are the most common risk factors associated with post-ERCP pancreatitis [3,4]. Visceral artery aneurysm (VAA) remains one of the rare complications associated with ERCP with associated sequelae. This may result from the manipulation of the pancreaticobiliary ductal system during sphincterotomy or stent placement, or it may occur following pancreatitis [5,6].

## Case presentation

**Figure 1:**
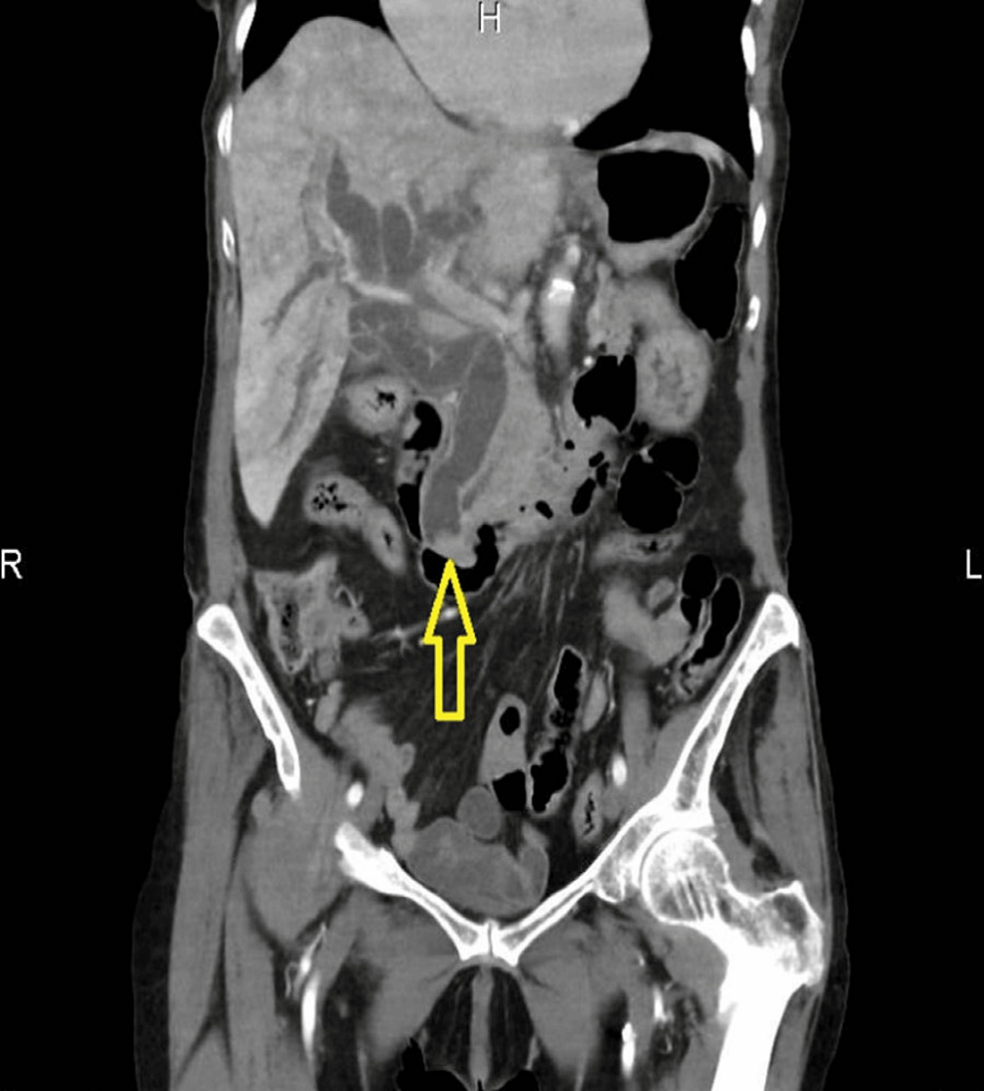
Coronal section of pre-ERCP CT showing dilated CBD and stone at the distal CBD (yellow open arrow). ERCP: endoscopic retrograde cholangiopancreatography; CT: computed tomography; CBD: common bile duct

A 93-year-old lady was brought into the emergency department (ED) of the hospital by ambulance following a fall at home. Initially, she had a fall two days ago in the kitchen when she noticed her right leg gave away. Following, she had a similar fall while coming out of bed and called the ambulance leading to her being escorted to the hospital. On arrival, she was found to be hemodynamically stable, but with obvious swelling of the right knee and unable to weight bear. She was reviewed by the orthopaedic team, and imaging suggested a large effusion in the suprapatellar pouch with severe tricompartmental osteoarthritis (OA). This was confirmed with the successful right knee joint aspiration, and analgesia was prescribed. She was also found on abdominal examination to have tenderness along the right hypochondrium with no guarding with deranged liver function tests (LFTs) and increased inflammatory markers. Computed tomography (CT) of the abdomen and pelvis showed biliary obstruction due to distal common bile duct (CBD) calculus (Figure 1). 

**Figure 2:**
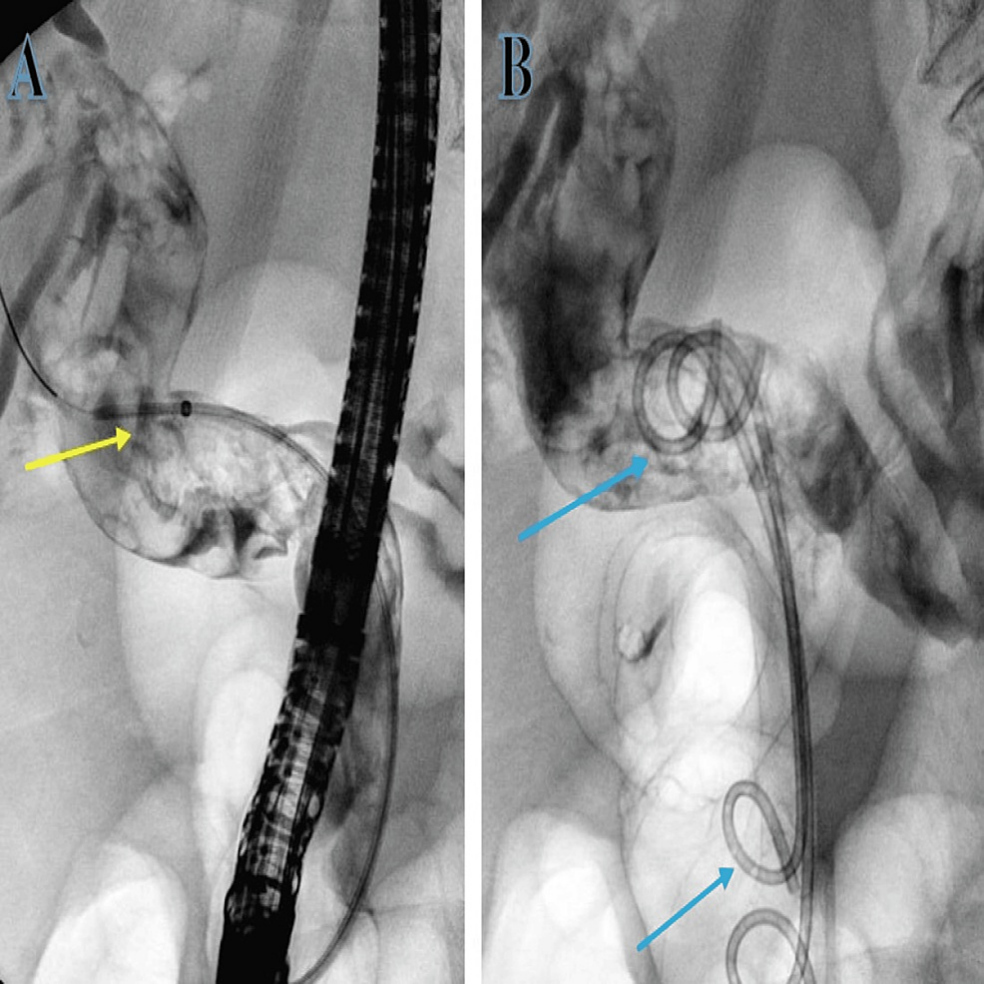
Deployment of pigtail stent with ERCP. (A) Guidewire inserted (yellow arrow). (B) Pigtail successfully deployed (blue arrows). ERCP: endoscopic retrograde cholangiopancreatography

The patient was started on broad-spectrum intravenous antibiotic coverage with piperacillin with tazobactam (Tazocin) for cholangitis and underwent an in-patient ERCP where CBD was noticed to be filled with blood clots during biliary cannulation, but no stones were seen. The clots were successfully evacuated with irrigation followed by the placement of two pigtail stents (Figure 2).

**Figure 3:**
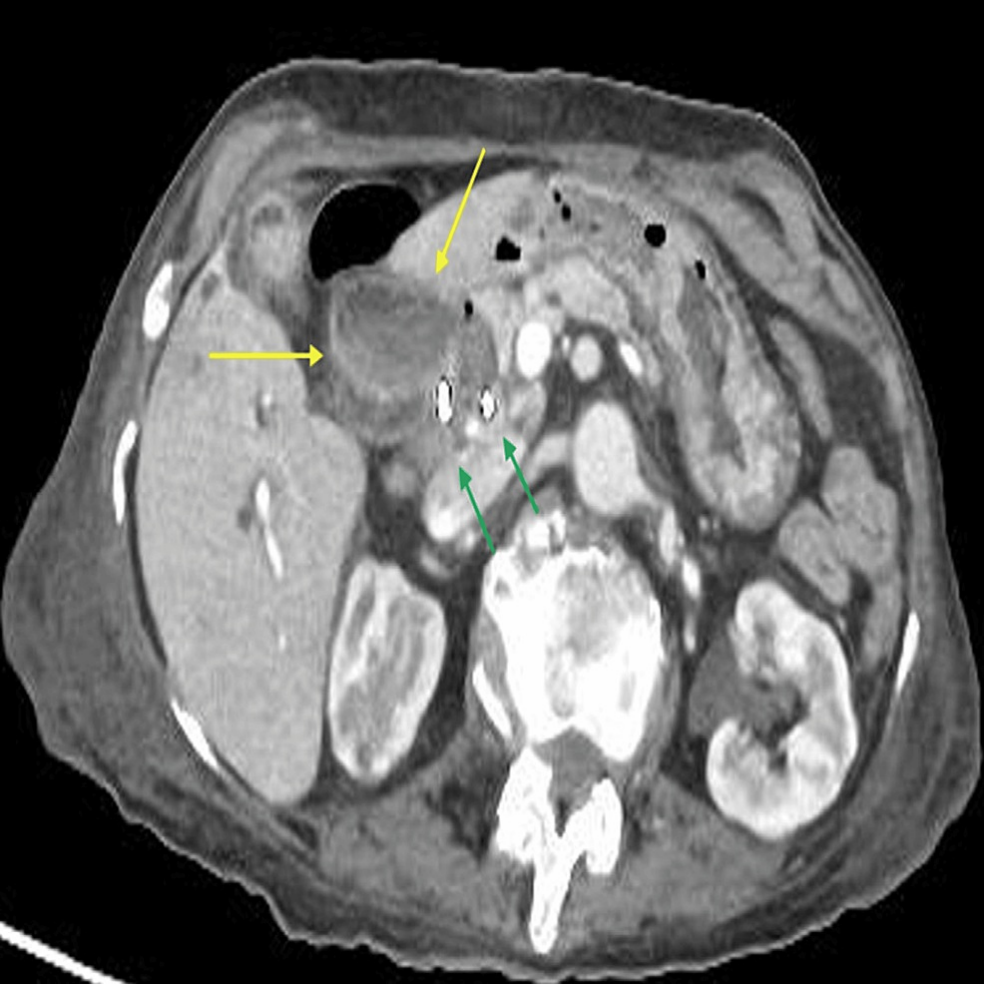
Abdominal CT axial image with high attenuated blood products along the intrahepatic and dilated biliary ducts (yellow arrows). Also seen are pigtail stents inserted previously during ERCP to help drainage (green arrows). CT: computed tomography; ERCP: endoscopic retrograde cholangiopancreatography

Since there were no stones found during ERCP and only clots were successfully evacuated with no concerns, an impression was made of stones that had spontaneously passed down the biliary tree. The initial blood culture grew *Escherichia coli* (*E. coli*), and in consultation with the microbiology team, the patient was prescribed intravenous meropenem and Tazocin based on sensitivities. Post-ERCP 15 hours later, she was reported by the nursing team to have per rectal bleeding with a drop in haemoglobin and persistently raised inflammatory marker, namely, C-reactive protein (CRP) of 25 milligrams per litre (mg/l) (reference range of 0-5 mg/l), and repeat blood culture grew vancomycin-resistant *Enterococcus* (VRE). In consultation with the microbiology team, her antibiotics were changed to linezolid and Tazocin. She had a repeat CT of the abdomen and pelvis that showed haemobilia (Figure 3).

**Figure 4:**
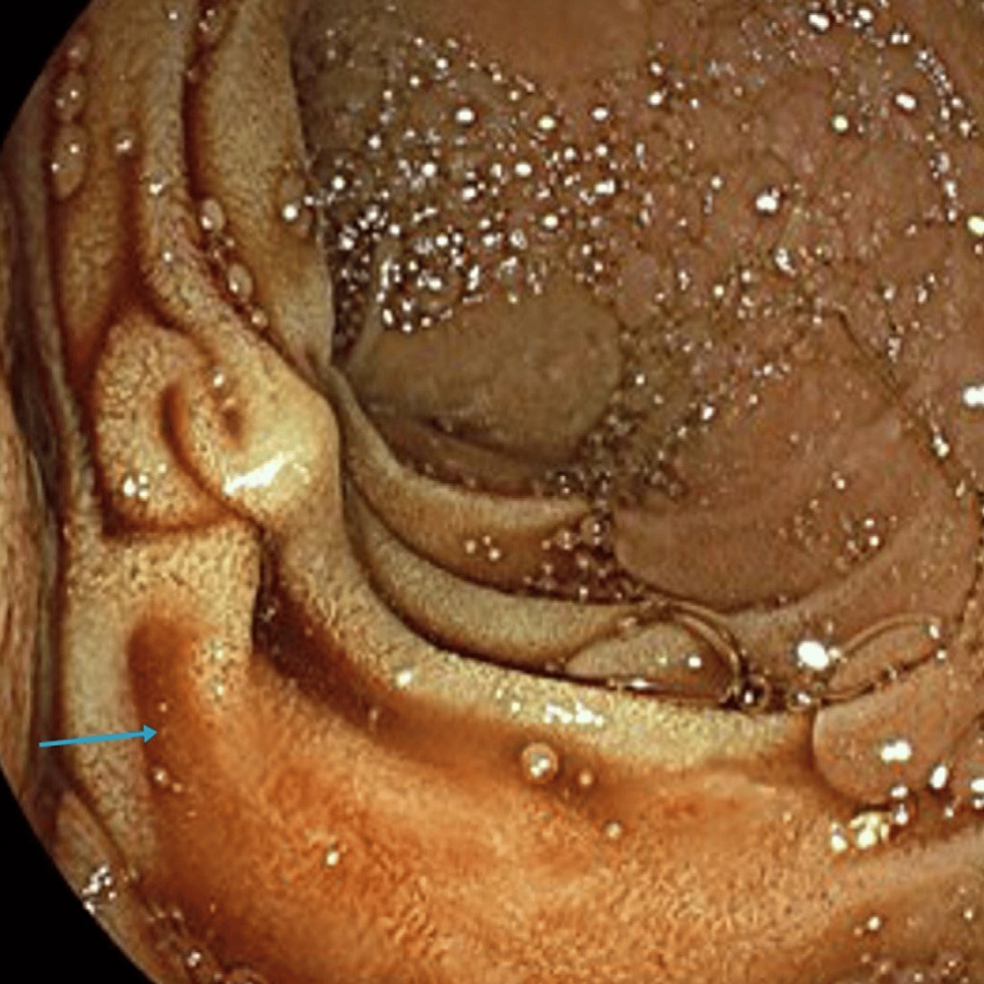
Duodenoscope along the ampulla post-suction of blood clots with a trace of remnant blood products in the duodenum (blue arrow).

The patient was transfused 2 units of red cell concentrate (RCCs), and an in-patient oesophagogastroduodenoscopy (OGD) was organised which showed blood in the duodenum that was successfully suctioned (Figure 4). 

She was started on intravenous proton pump inhibitors (PPI) and underwent a CT of the abdomen and pelvis along the coeliac-mesenteric axis that suggested a 1.5 cm pseudoaneurysm arising from one of the segmental branches of the hepatic artery along segment 2 of the liver.

**Figure 5:**
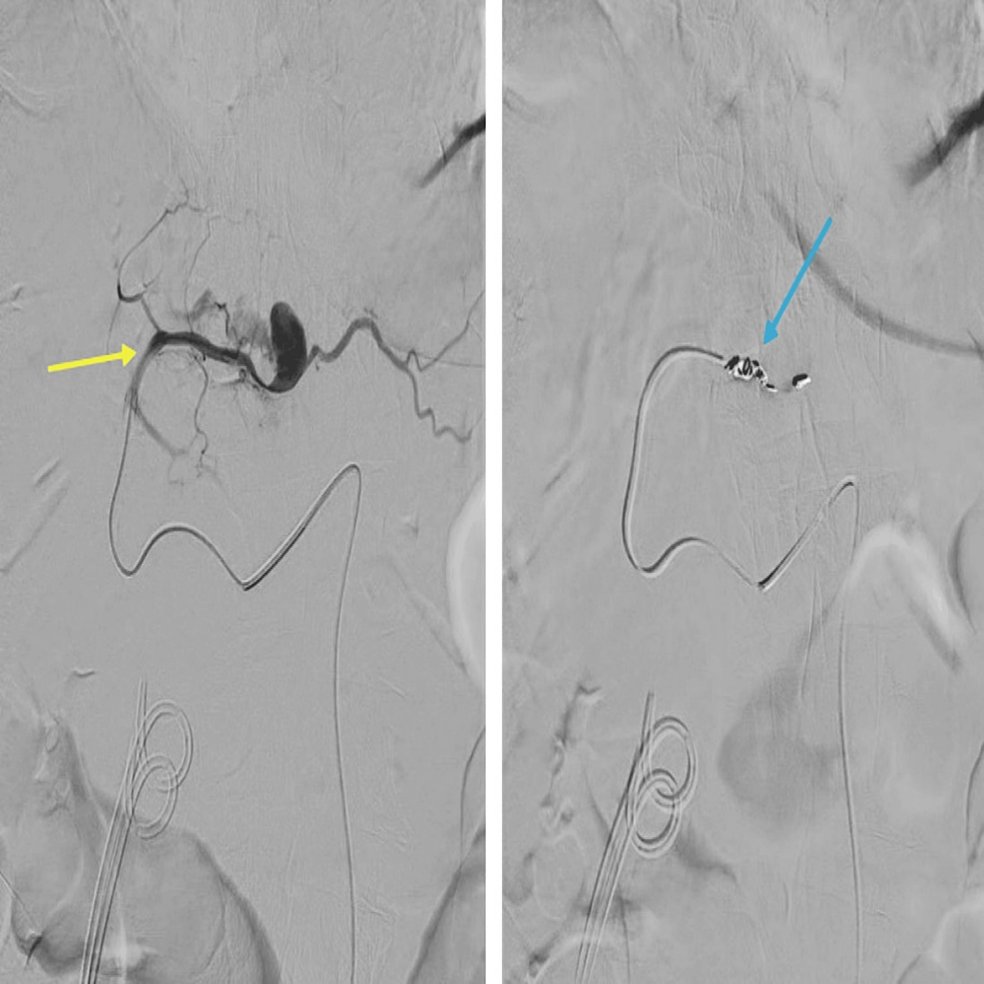
(A) Selective left hepatic angiogram along the feeding vessel with pseudoaneurysm (yellow arrow). (B) Hepatic arteriogram showing successful coil embolization of the segmental branch of the hepatic artery along segment 2 of the liver with 4 mm coils with no filling defects post-embolization (blue arrow).

The patient underwent embolization of the left hepatic artery pseudoaneurysm by the interventional radiology team via a retrograde right common femoral artery (CFA) puncture. The CT angiogram along the coeliac-mesenteric axis confirmed a pseudoaneurysm from the segmental branch of the hepatic artery along segment 2 of the liver. This was selectively cannulated with a prograde catheter. The feeding vessel was successfully embolized with 4 mm coils, and no filling of the pseudoaneurysm was seen post-embolization (Figure 5).

Following blood tests, a day later showed normalisation of the infection markers and blood count. The physiotherapy team reviewed the patient, who was transferred to the rehabilitation ward and eventually discharged back to the community to the care of the general practitioner (GP). 

## Discussion

The association of bleeding with combined ERCP and sphincterotomy has been reported to occur in up to 2% of patients with a prevalence of severe bleeding being 0.1-0.5% [7,8].

Vascular arterial aneurysms include true aneurysms stemming from the intrinsic vessel wall defect or a pseudoaneurysm arising from the manipulation of the pancreaticobiliary duct with duodenoscope during sphincterotomy or post-inflammation due to pancreatitis [9,10].

Pancreaticoduodenal and gastroduodenal artery aneurysms together account for only 3.5% of all aneurysms, while pancreatitis has been reported to be associated with aneurysms in almost half of the cases [11,12]. Pseudoaneurysm due to pancreatitis could stem from the direct enzymatic degradation of the vascular walls [13]. Another risk factor for the formation of pseudoaneurysm is cholangitis in patients who underwent cholecystectomy [14]. Li et al. have reported a patient who had undergone open cholecystectomy and developed cholangitis and pancreatitis which they suggest as precipitant towards the formation of pseudoaneurysm post-ERCP [15]. In our patient, there was no preceding cholecystectomy, and although he did have cholangitis that was managed with intravenous antibiotics, he did not develop pancreatitis. Li et al. concluded following the endoscopic and angiographic reviews that perhaps it was the guidewire-related injury within the left hepatic duct penetration that may have led to the ERCP-related formation of pseudoaneurysm and bleeding, as CT and magnetic resonance cholangiopancreatography (MRCP) imaging performed before ERCP did not disclose any hematoma or pseudoaneurysm [15]. 

Briefly, another reported reason for bleeding following ERCP in literature (although not in the case of our patient here) is the phenomenon of post-sphincterotomy bleeding managed with options including balloon tamponade, injection of diluted adrenaline of strength of 1:10,000, or spray irrigation [16]. The application of monopolar electrocautery in the vicinity of the duodenal papilla while avoiding the opening of the pancreatic duct at the 5 o'clock position is another mode for the management of post-ERCP bleeding [17]. The application of endoscopic clips with a duodenoscope is yet another mode of securing hemostasis. However, it is reported to be associated with technical difficulties with clip deployment as the plastic sheath may bend or get kinked while passing over the elevator located at the bottom of the instrument channel [16]. Although in some patients spontaneous thrombotic obstruction of the pseudoaneurysm has been reported [6], the interventional radiology-guided angiogram followed by the embolization of the pseudoaneurysm bleeding remains the choice of treatment due to the diagnostic precision and instant deliverance of the therapeutics [18]. The availability of less traumatic guidewires has now improved the avoidance of duodenoscope-related haemobilia [19], although in patients with persistent bleeding, surgery remains the last viable option [20]. Other recommendations to avoid pseudoaneurysm bleeding are to avoid going too far in the biliary tree with the guidewire during biliary cannulation [15].

Although there remains a limited possibility that our patient who did not have prior cholecystectomy may have suffered from the bleeding from the segmental branch of the hepatic artery pseudoaneurysm following biliary cannulation, it may have also resulted from cholangitis itself. Lastly, it could also be argued that perhaps the pseudoaneurysm may have been present beforehand as clots were seen during ERCP, although this is excluded from the CT done before ERCP that did not show any pseudoaneurysms.

It would be interesting to see more case reports to understand the phenomenon more decisively.

## Conclusions

This case report highlights the importance of understanding the side effects and association with inflammation along the biliary ducts and pancreas whereby bleeding during the procedure or delayed post-procedural bleeding may occur. An understanding of prior imaging reviews to rule out any aneurysmal dilatation remains vital. Prompt diagnosis using imaging modalities with interventional radiology-guided embolization is the appropriate mode of management. The case report also suggests that although post-traumatic pseudoaneurysm bleeding has been reported, this may also occur due to inflammation along the hepatobiliary region.
